# Are Koreans Prepared for the Rapid Increase of the Single-Household Elderly? Life Satisfaction and Depression of the Single-Household Elderly in Korea

**DOI:** 10.1155/2013/972194

**Published:** 2013-10-30

**Authors:** Mi-Ra Won, Yun-Jung Choi

**Affiliations:** ^1^Department of Nursing, Daewon University College, 316 Daehak-Road, Jecheon-City, Chungbuk 390-702, Republic of Korea; ^2^Red Cross College of Nursing, Chung-Ang University, 221 Heukseok-Dong, Dongjak-Gu, Seoul 156-756, Republic of Korea

## Abstract

*Purpose*. In Korea, it has been estimated that the number of the single-household elderly increased 45% from 2005 to 2010. This research was conducted to provide empirical resources for development of a community mental health program by an explorative investigation on depression, coping mechanism, and life satisfaction of a single-household elderly population. *Design and Methods*. This research applied a descriptive survey research design. Participants were 225 single-household elderlies residing in Seoul, Korea. The geriatric depression scale and the satisfaction with life scale were used to check the level of depression and life satisfaction of the participants. *Results*. Results showed that 46.3 percent of the participants were categorized as having light-to-severe level of depression, and 80.5 percent of the participants responded that they were dissatisfied with their lives. This research demonstrated that the level of depression and life satisfaction of the Korean single-household elderly is statistically significantly related to age and gender as well as coping resources and human resources. *Implications*. Current public health services in Korea for the single-household elderly are still lacking and require active support, intervention, and research to provide effective programs and services. Case management, counseling, and various programs based on Korean culture including support from family members and community-based assistance are recommended to help the vulnerable population.

## 1. Introduction

Since 2000, Korea has become an aging society, and the proportion of the population considered to be aging has accelerated from 3.8% in 1980 to 11% in 2010. Estimates show that the proportion of the elderly will geometrically progress to 24.3% in 2030 and 37.4% in 2050. Among single-households, the proportion involving the elderly household was 19.2% in 2010, which accounted for the largest portion. It has been estimated that the number of single-household elderlies increased 45% from 2005 to 2010 [1].

Factors related to the elderly life satisfaction include adaptation from losses such as physical degeneration, retirement, reduced income, and death of spouse or friends [2]. A recent study [3] reported that, among the Korean elderly, approximately 20% experienced depression which interfered with their daily lives and which continued for more than 2 weeks. Depression in old age is a threat to psychiatric as well as physical health, which deteriorates the quality of life [4]. Elderly people who live alone are placed in risky mental health conditions; it has been reported that 15% used alcohol and 60% had an alcohol use problem, and their level of suicidal ideation was higher than that of general population [5]. 

Life satisfaction is a factor which is directly related to depression in the elderly segment of the population. Life satisfaction of the elderly residing alone is lower than that of the elderly residing with family members; specifically in case of elderly women, the level of satisfaction is lower than that of the population living with their spouses [4, 6]. Coping mechanisms for senile depression which include activities regarding production or recreation improve mental health by enhancing the ability of environment control. Human resources of the elderly reinforce their coping mechanisms that maintain their psychological wellbeing [7]. 

There is a pressing need to focus on the single-household elderly and to develop social support network for them [2, 4, 6]. This research was conducted to provide empirical data for developing a community mental health program. The present study was an explorative investigation on depression, coping mechanism, and life satisfaction of the single-household elderly population.

## 2. Methods

### 2.1. Participants

The research participants were recruited from a community mental health center in Seoul, Korea. Staffs from the mental health center visited every single-household elderly person in the community who was over 65 years of age and had not been diagnosed with psychiatric disorders. Initially, 260 elderlies were included in the study. They were provided with a clear and sufficient explanation regarding the research purposes and process. Those who subsequently voluntarily agreed to participate were included. The total number of the participants was 225 single-household elderlies. All of the participants were informed of their rights to withdraw from the study at any time without fear of being penalized. A participant was not required to respond if he or she did not wish to respond. This research received an approval from the institutional review board (IRB), and the participants' personal information was kept confidential. 

### 2.2. Instruments

#### 2.2.1. K-GDS

The Korean geriatric depression scale (K-GDS) was used to check the level of depression, which was originally developed by Yesavage et al. [8] and translated into Korean by Jung et al. [9]. It contains 30 items, and a higher score indicates a higher level of depression. The Cronbach's alpha was from 0.86 to 0.94 [10, 11].

#### 2.2.2. K-SWLS

To test the level of life satisfaction, the Korean satisfaction with life scale (K-SWLS) originally developed by Diener et al. [12] was translated into Korean by Ryu [13]. It consisted of five questions, with a higher score indicating greater level of life satisfaction. The Cronbach's alpha was from 0.82 to 0.85 [14, 15].

### 2.3. Data Analysis

Collected data was analyzed by the SPSS/WIN 15.0 pc program. Demographic characteristics of the participants, K-GDS and K-SWLS, and coping resources and human resources were calculated by descriptive statistics. K-GDS and K-SWLS by demographic characteristics, coping resources, and human resources were analyzed by means of Student's *t*-test and ANOVA. 

## 3. Results

The mean age of the research participants was 76.3 years and ranged from 67 to 92 years (Table 1). Over 60% of the participants were in their seventies, and two-third of the participants were women (72.4%). The level of depression evaluated by K-GDS indicated that 46.3% of the participants were categorized as having light-to-severe level of depression, while the mean score of the K-GDS demonstrated that they were in the margin of the normal range (13.36/30). The mean score of K-SWLS demonstrated that the level of life satisfaction was 15.4/25, indicative of “a little dissatisfied.” Most (80.5%) of the participants responded that they were dissatisfied with their lives. 

 The surveyed elderly used coping resources when they felt depressed included watching television (48.9%), meeting friends (14.2%), religious activities (9.8%), and physical activity (7.1%). But, 21.8% had no coping resources. Human resources of the participants were family members (33.8%), religious organizations (11.1%), and public organizations (3.1%). But, 36.5% had no human resources. 

Factors related to the level of depression were gender (*t* = 2.12, *P* = 0.035), coping resource (*t* = −3.16, *P* = 0.002), and human resource (*t* = −7.17, *P* = 0.000), while age was not significantly related to the level of depression (*F* = 1.64, *P* = 0.182) (Table 2). The reports indicated that the levels of depression differed from presence of coping resources and human resources, as well as gender of the elderly. The level of life satisfaction was related to gender (*t* = −4.05, *P* = 0.000), age (*F* = 3.73, *P* = 0.012), and coping resource (*t* = 3.86, *P* = 0.000). The results suggested that the levels of life satisfaction varied with presence of coping resources, age, and gender of the elderly.

## 4. Discussion

The demographic characteristics of this research demonstrate a greater proportion of single-household elderly women than men, echoing previous studies [2, 5]. This difference of gender distribution needs to be considered to maintain a balance in developing programs for the single-household elderly. For example, characteristics and preference of elderly women will need to be reflected in the program, while respecting the minority of elderly men.

Approximately, half of the participants (46.3%) of this research indicated “depression,” which was a similar level of distribution as previous surveys [7], while showing a higher level of depression than that in the study of Kim et al. [16]. Although the single-household elderly display a higher level of depression, their coping resources and human resources are insufficient. At present, about half of the participants reported “watching television” as their coping method, 40% of the participants had no human resources when they experienced depression, and 80.5% opined being dissatisfied with their lives. These conditions are in line with the study of Sok [6] which reported that the elderly who live alone display less family support and life satisfaction than those living with own family members. Depression of the single-household elderly deteriorates their quality of life, which can precipitate physical as well as mental health detriments, which in turn raise social expenses. Suicidal risk is also increased, requiring intensive and active intervention.

In this research, demographic characteristics of the participants that affected depression, life satisfaction, and human resources were examined. There was a difference between gender and human resources, and elderly men were more depressed and less satisfied with their lives than elderly women. The data support the suggestion that elderly males are a more vulnerable population than single-household elderly women. Generally, women are more competent to make and maintain relationships with others [4] and more proficient to get public assistance than men [2]. Elderly males are most at risk for attempted suicide [17]. Therefore, in caring for the elderly, single-household elderly males need great consideration. 

Results of this research showed that age was another factor among the demographic characteristics which affected life satisfaction of the participants. Life satisfaction of the elderly in this research increased with age. The result is not consistent with reports from Kim and Pyo [7], which stated that the older the participants were, the more satisfied they were with their lives. Generally, with increased age, social activities decrease and the level of depression increases [18]. It can be hypothesized that life satisfaction among the elderly population is regained as aging with the mechanism of adaptation to the multiple changes in life associated with aging. 

In this research, the level of depression of the participants was related to life satisfaction, coping resources, and human resources. The more dissatisfied the elderly were with their lives, the more severe was their depression and the less available for human resources were they. Those results are in line with previous studies which reported that life satisfaction, depression, and social support are strongly interrelated to each other [2, 16]. In Korean culture, the elderly are respected and cared for emotionally, economically, and by familial social support [17]. Thus, the relationship of the Korean elderly with their children is crucial as basic coping resources and human resources which affect life satisfaction and depression, even when the elderly person resides alone [4]. Reinforcement of family relationships and support must be considered in developing a program for the single-household elderly. Facilitating participation of family members and friends would be helpful to enhance the life satisfaction and to prevent depression of the elderly. 

## 5. Conclusion

As the societal proportion of the elderly continues to increase, the collective mental health of the population is being compromised. Depression of the single-household elderly deteriorates their quality of life and increases their risk of suicidal behavior, which warrants public consideration and assistance. Current public health services in Korea for the population are still lacking and require active support, intervention, and research to provide effective programs and services. Case management, counseling, and various programs including support from family members and community-based assistance are recommended to help the vulnerable population. 

## Figures and Tables

**Table 1:** Characteristics and variables of subjects (*N* = 225).

Variables	Categories	*N* (%)	Min.	Max.	Mean	SD
Gender	male	62 (27.6)				
	female	163 (72.4)				

Age (years old)	65–70	36 (16.0)	67	92	76.32	0.66
	71–80	136 (60.5)				
	81–90	50 (22.2)				
	90<	3 (1.3)				

GDS	normal	121 (53.7)	5	30	13.36	1.21
	mild depression	40 (17.8)				
	moderate depression	17 (7.6)				
	severe depression	47 (20.9)				

SWLS	very dissatisfied	33 (14.7)	5	30	15.04	1.13
	dissatisfied	57 (25.3)				
	little dissatisfied	91 (40.5)				
	moderate	27 (12.0)				
	little satisfied	14 (6.2)				
	satisfied	3 (1.3)				
	very satisfied	0 (0.0)				

Coping resource	have	174 (77.3)				
	lack	49 (21.8)				

Human resource	have	142 (63.1)				
	lack	82 (36.5)				

**Table 2:** GDS and SWLS by multiple comparisons.

Variables	Categories	GDS			SWLS		
		Mean	SD	*t* or *F*	Mean	SD	*t* or *F*
Gender	male	15.97	7.14	2.12 (0.035*)	13.00	5.12	−4.05 (0.000**)
	female	13.74	6.99		15.82	4.47	

Age (years old)	65–70	2.11	1.24	1.64 (0.182)	2.19	0.95	3.73 (0.012*)
	71–80	1.90	1.19		2.80	1.14	
	81–90	1.90	1.22		2.96	1.12	
	90<	3.33	1.16		2.67	0.58	

Coping resource	have	13.62	6.88	−3.16 (0.002*)	15.01	4.83	−0.03 (0.970)
	lack	17.18	7.23		15.04	4.85	

Human resource	have	12.05	6.09	−7.17 (0.000**)	15.94	4.78	3.86 (0.000**)
	lack	18.42	6.92		13.43	4.48	

*P < 0.05; **P < 0.001.
